# Oxygen and Heterotrophy Affect Calcification of the Scleractinian Coral *Galaxea fascicularis*

**DOI:** 10.1371/journal.pone.0052702

**Published:** 2012-12-21

**Authors:** Tim Wijgerde, Saskia Jurriaans, Marleen Hoofd, Johan A. J. Verreth, Ronald Osinga

**Affiliations:** Aquaculture and Fisheries Group, Department of Animal Sciences, Wageningen University, Wageningen University and Research Centre, Wageningen, The Netherlands; Leibniz Center for Tropical Marine Ecology, Germany

## Abstract

Heterotrophy is known to stimulate calcification of scleractinian corals, possibly through enhanced organic matrix synthesis and photosynthesis, and increased supply of metabolic DIC. In contrast to the positive long-term effects of heterotrophy, inhibition of calcification has been observed during feeding, which may be explained by a temporal oxygen limitation in coral tissue. To test this hypothesis, we measured the short-term effects of zooplankton feeding on light and dark calcification rates of the scleractinian coral *Galaxea fascicularis* (n = 4) at oxygen saturation levels ranging from 13 to 280%. Significant main and interactive effects of oxygen, heterotrophy and light on calcification rates were found (three-way factorial repeated measures ANOVA, p<0.05). Light and dark calcification rates of unfed corals were severely affected by hypoxia and hyperoxia, with optimal rates at 110% saturation. Light calcification rates of fed corals exhibited a similar trend, with highest rates at 150% saturation. In contrast, dark calcification rates of fed corals were close to zero under all oxygen saturations. We conclude that oxygen exerts a strong control over light and dark calcification rates of corals, and propose that *in situ* calcification rates are highly dynamic. Nevertheless, the inhibitory effect of heterotrophy on dark calcification appears to be oxygen-independent. We hypothesize that dark calcification is impaired during zooplankton feeding by a temporal decrease of the pH and aragonite saturation state of the calcifying medium, caused by increased respiration rates. This may invoke a transient reallocation of metabolic energy to soft tissue growth and organic matrix synthesis. These insights enhance our understanding of how oxygen and heterotrophy affect coral calcification, both *in situ* as well as in aquaculture.

## Introduction

It is well established that coral calcification, the precipitation of aragonite from calcium and carbonate ions by scleractinian corals, is stimulated by heterotrophy ([1] and references therein). The positive effect of heterotrophy on calcification is thought to be mediated through enhanced organic matrix synthesis [2], [3], [4], increased photosynthesis rates [4], [5], [6], [7], [8], [9] and increased supply of metabolic DIC [3], [10]. Although the enhancement of coral calcification by heterotrophy has been demonstrated with long-term experiments [1], little is known about the short-term effects of feeding. In fact, heterotrophy has been shown to have a short-term inhibitory effect on dark calcification rates [11], [12]. This discrepancy is not well understood. Several authors have stated that in darkness, inhibition of calcification during zooplankton, glycerol or glucose supplementation may be caused by a temporal reallocation of energy, for example to prey capture and nutrient uptake [11], [12]. This reallocation of energy in darkness may involve a temporal decrease in tissue oxygen concentrations during prey capture and nutrient uptake, without photosynthetic oxygen production to compensate for this. As oxygen is a prerequisite for ATP-synthesis through oxidative phosphorylation in calicoblastic mitochondria [13], oxygen limitation may result in impaired ATP production and, hence, impaired calcification rates, as Ca^2+^/H^+^ ATP-ases require ATP or ADP for active transport of calcium ions and protons over the calicoblastic membrane [14]. Indeed, Rinkevich and Loya [15] and Colombo-Pallotta et al. [12] found that external oxygen supplementation enhances dark calcification rates of *Stylophora pistillata* and *Montastraea faveolata*, respectively, supporting the theory that oxygen limitation may indeed impair dark calcification of scleractinian corals during feeding.

In this study, we aimed to improve upon the current model of coral calcification by determining the combined effects of dissolved oxygen and heterotrophy on calcification. To this end, we measured light and dark calcification rates of the scleractinian coral *Galaxea fascicularis* with and without zooplankton supplementation under a range of ambient oxygen saturations. Profound interactive effects of oxygen, heterotrophy and light were found, demonstrating that these factors exert a strong control over coral calcification.

## Results

**Figure 1:**
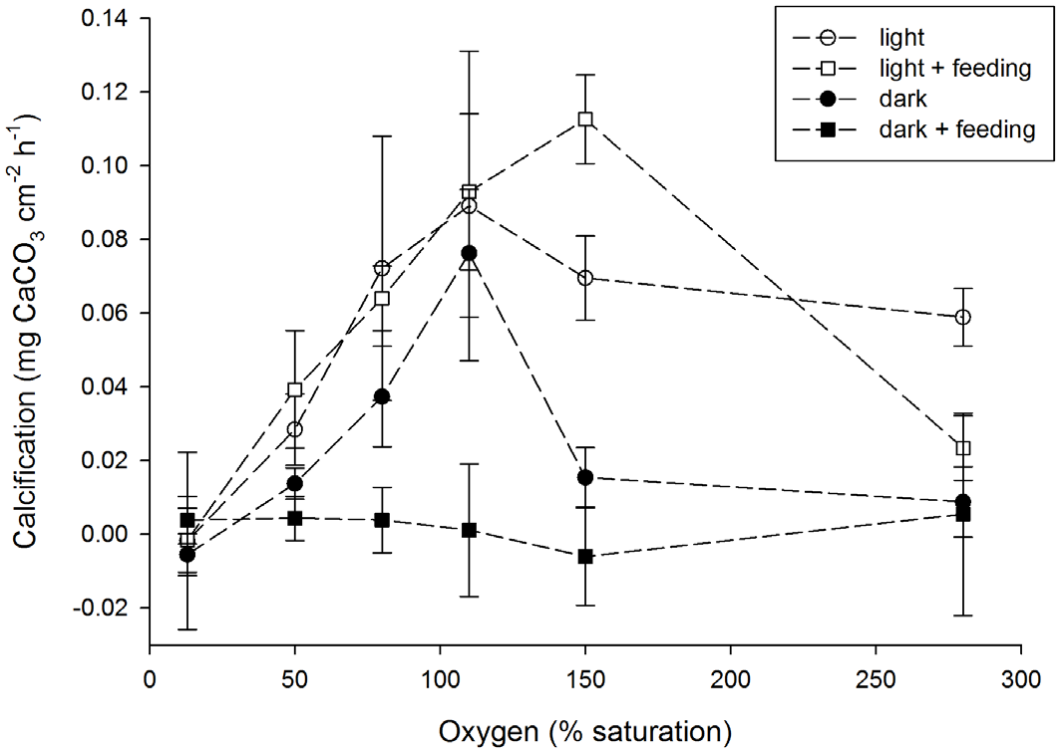
Effects of oxygen and heterotrophy on light and dark calcification of *Galaxea fascicularis*. Feeding quantity was 150 *Artemia* nauplii polyp^−1^. QI in light was 250 µmol m^−2^ s^−1^. Values are means ± s.d. (n = 4).

*G. fascicularis* colonies exhibited highly variable calcification rates between treatments, ranging from −0.006±0.006 to 0.113±0.012 mg CaCO_3_ cm^−2^ h^−1^ (Figure 1). At the end of all feeding treatments, coral polyps exhibited a distinct feeding response, reflected by extrusion of mesenterial filaments which enveloped *Artemia* aggregates (not shown). Light and dark calcification rates of unfed corals were clearly affected by oxygen. In light, calcification rates were negative at 13%, impaired at 50 and 80%, optimal at 110%, and inhibited at 150 to 280% saturation. Dark calcification rates exhibited a similar pattern, where calcification impairment was highly pronounced at 150 and 280% saturation.

Corals fed with zooplankton exhibited a different trend. Light calcification rates of fed corals were negative at 13% saturation, impaired at 50 and 80%, optimal at 150% oxygen saturation, and considerably inhibited at 280% saturation. In contrast, dark calcification rates of fed corals were close to zero under all oxygen saturations.

Statistical analysis revealed that oxygen, heterotrophy and light exerted main and/or interactive effects on calcification rates (Table 1). Oxygen had a significant effect on calcification (F_1.379,4.138_ = 21.009, p = 0.008, Table 1), where overall calcification rates were significantly higher at 80, 110 and 150% oxygen saturation compared to 13% (Bonferroni, p = 0.039, p = 0.020 and p = 0.038, respectively), irrespective of light conditions and feeding. At 150% saturation, overall calcification was also significantly higher compared to 50% (Bonferroni, p = 0.015).

**Table 1:** Main and interactive effects of oxygen, heterotrophy and light on *G. fascicularis* calcification.

Factor	Variable	F	df	p
	calcification			
Oxygen		21.009	1.379	0.008*
Heterotrophy		2.207	1	0.234
Light		38.597	1	0.008*
Oxygen*Heterotrophy		10.386	2.014	0.011*
Oxygen*Light		13.339	2.207	0.004*
Light*Heterotrophy		18.380	1	0.023*
Oxygen*Heterotrophy*Light		15.350	2.557	0.002*

* Indicates significant effect (p<0.050), three-way factorial ANOVA for repeated measures (n = 4).

A significant main effect of light on calcification rates was also found (F_1,3_ = 38.597, p = 0.008, Table 1), where overall light calcification rates were significantly higher compared to those in darkness, irrespective of oxygen saturation and feeding.

There was no significant main effect of heterotrophy on calcification rates (F_1,3_ = 2.207, p = 0.234, Table 1), hence in general, calcification rates of fed corals were not different from unfed corals, irrespective of oxygen saturation and light.

Light and heterotrophy exhibited a significant interactive effect on calcification rates (F_1,3_ = 18.380, p = 0.023, Table 1), irrespective of oxygen saturation. This was reflected by the fact that feeding inhibited calcification in darkness but not in light (simple effect, F_1,3_ = 26.510, p = 0.014 and F_1,3_ = 0.070, p = 0.815, respectively), irrespective of oxygen saturation.

A significant interactive effect of oxygen and heterotrophy on calcification rates was also found F_2.014,6.043_ = 10.386, p = 0.011, Table 1). This was reflected by the fact that heterotrophy had no effect on calcification rates except at 150% oxygen saturation, at which calcification was enhanced (simple effect, F_1,3_ = 12.800, p = 0.037), irrespective of light conditions.

Oxygen and light exhibited a significant interactive effect on calcification rates (F_2.207,6.620_ = 13.339, p = 0.004, Table 1). This was reflected by the fact that light enhanced calcification at all oxygen saturations except at 13% (simple effect, F_1,3_ = 0.020, p = 0.887), irrespective of feeding.

Finally, there was a significant interactive effect of oxygen, light and heterotrophy on calcification rates (F_2.557,7.672_ = 15.350, p = 0.002, Table 1). This was reflected by a different interaction between oxygen and heterotrophy in light compared to darkness. More specifically, in light, feeding had no effect on calcification rates at 13% (simple effect, F_1,3_ = 0.150, p = 0.723), 80% (F_1,3_ = 3.480, p = 0.159) and 110% oxygen saturation (F_1,3_ = 0.570, p = 0.506), a positive effect at 50 and 150% (F_1,3_ = 19.31, p = 0.022 and F_1,3_ = 55.29, p = 0.005, respectively) and an inhibitory effect at 280% (F_1,3_ = 34.94, p = 0.010). In darkness, however, feeding had an inhibitory effect at oxygen saturations of 50% (F_1,3_ = 30.94, p = 0.011), 80% (F_1,3_ = 104.27, p = 0.002), 110% (F_1,3_ = 27.08, p = 0.014) and 150% (F_1,3_ = 103.83, p = 0.002) and no effect at extreme saturations of 13 and 280% (F_1,3_ = 0.780, p = 0.441, F_1,3_ = 2.65, p = 0.202, respectively).

## Discussion

*G. fascicularis* exhibited highly variable calcification rates between treatments, which lie in the same range as found for the scleractinian coral *Montastraea faveolata* [12]. This study revealed significant main and interactive effects of oxygen, heterotrophy and light on calcification rates of the scleractinian coral *Galaxea fascicularis*, demonstrating that these factors affect calcification in a complex manner.

First of all, significant main and interactive effects of oxygen were found. Overall calcification rates were highest at 80, 110 and 150% oxygen saturation, irrespective of light conditions and zooplankton feeding. At lower saturations of 13 and 50%, overall calcification rates were significantly impaired. This observation suggests a limiting role of oxygen in the calcification process. Not only did higher oxygen saturations initially promote overall calcification rates, at 110% saturation, dark calcification rates of unfed corals were not significantly different from those in light. This is in accordance with the findings of Rinkevich and Loya [15] and Colombo-Pallotta et al. [12], who found that oxygen enhances dark calcification rates of *Stylophora pistillata* and *Montastraea faveolata*, respectively. The causal mechanism behind the enhancement of light and dark calcification by oxygen may involve augmented ATP production through increased oxidative phosphorylation inside calicoblastic mitochondria, subsequently promoting Ca^2+^/H^+^ ATP-ase activity [14], [16]. Apparently, this oxygen effect is more important than other proposed mechanisms underlying light enhanced calcification, most notable regulation of tissue pH by photosynthesis [10], [17]. Interestingly, light calcification rates of fed and unfed corals were also impaired by hypoxia, which may be explained by a significant efflux of oxygen to the surrounding water. At low ambient oxygen saturations, a high oxygen gradient between gastrodermal cells harbouring photoautotrophic zooxanthellae and the surrounding water (approx. 240 versus 13 to 80% saturation [18]) may have induced high oxygen efflux rates via the coelenteron, at the expense of the calicoblastic cells. As the incubation chambers were provided with ample water flow, this phenomenon is likely to have been further enhanced as flow enhances oxygen efflux from coral tissue in light [19]. This may have resulted in oxygen depletion of calicoblastic cells and a subsequent calcification impairment during the hypoxia treatments.

The inhibition of light and dark calcification rates at 280% saturation suggests oxygen intoxication. The toxic effect of hyperoxia on cells and organisms is well-known, and is caused by the formation of reactive superoxide radicals (O_2_^−^), i.e. oxygen molecules with one or more unpaired electrons [20]. In corals, such a hyperoxic environment is generated intracellularly by photosynthetic activity of zooxanthellae and xanthine oxidase [18], [21]. Although the coral holobiont uses superoxide dismutases, catalase and ascorbate peroxidase to eliminate superoxide radicals [21], [22], [23], [24], these antioxidants may become overwhelmed at high oxygen levels [20]. This may have occurred during light and dark incubations at 280% oxygen saturation, resulting in (calicoblastic) cellular damage and a subsequent inhibition of calcification. In light, cellular damage may have impaired photosynthesis as well, and as photosynthesis is a major driver of calcification [16], [25], this may have contributed to the observed reductions in light calcification.

The pronounced inhibition of dark calcification by hypoxia, and the impairment of light calcification by hyperoxia has implications for our understanding of *in situ* calcification rates. Corals inhabiting lagoons and reef flats regularly experience hypoxia and hyperoxia due to minimal water flow rate and exchange during low tide, resulting in oxygen saturations ranging from approximately 30 to 194% [26], [27]. This suggests that corals may have highly variable calcification rates throughout the day and night, especially on reefs that experience low tides accompanied by low water flow. In addition, the coral-water interface becomes anoxic (approximately 1% saturation) during night time [28], [29], and hyperoxic (up to 373% saturation) during the day [29]. This suggests that corals may have highly variable calcification rates throughout the day and night, especially on reefs that experience low tides accompanied by low water flow. These daily oxygen dynamics should be taken into consideration when measuring reef accretion.

A significant main effect of light was also found, in accordance with the hypothesis of light-enhanced calcification [16], [25], as overall calcification rates were significantly higher in light compared to darkness, irrespective of oxygen saturation and zooplankton feeding. However, this main effect was in large part due to low dark calcification rates of fed corals. The enhancement of calcification by light was likely caused by intracellular oxygen production [18] and elevated tissue pH [10], [17] resulting from photosynthesis. As oxygen supplementation significantly enhanced dark calcification rates of unfed corals, the former process may have been most relevant.

Next to oxygen and light, heterotrophy had a pronounced interactive effect on calcification. Zooplankton feeding inhibited calcification in darkness but not in light, irrespective of oxygen saturation. We initially hypothesized that under dark conditions, the causal inhibitory mechanism of heterotrophy involves temporal oxygen limitation of calcifying calicoblastic cells, which could result in depletion of the intracellular ATP pool and a subsequent reduction of Ca^2+^/H^+^ ATP-ase activity [13], [14]. The three-way interaction, however, reveals that oxygen only promoted calcification rates of fed corals under light conditions at 50 and 150% oxygen saturation, which may be explained by increased oxygen demand during feeding. In darkness, oxygen was unable to alleviate the inhibitory effect of feeding on calcification rates. This strongly suggests that oxygen limitation is not the causal mechanism underlying inhibition of dark calcification by heterotrophy, even though oxygen demand may be higher in darkness.

**Figure 2:**
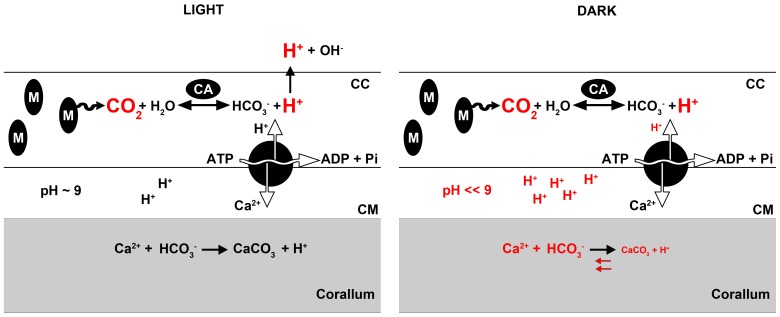
Conceptual model of dark calcification impairment by heterotrophy. Feeding increases metabolic rates, CO_2_ production, and as a result proton production in calicoblastic cells. In light, these protons are titrated by photosynthetically generated hydroxide ions in the coelenteron. In darkness, protons accumulate in the calicoblastic ectoderm, increasing the proton gradient between the calicoblastic ectoderm and the calcifying medium (CM). This causes a temporary decrease of the CM pH and aragonite saturation state, shifting the calcification reaction to the left. CC: calicoblastic cell. CM: calcifying medium. M: mitochondrion. CA: carbonic anhydrase. Model based on [10] and [17].

An alternative mechanism for the short-term inhibitory effect of heterotrophy on dark calcification may involve increased respiration rates, resulting in a temporary decrease of tissue pH levels through the conversion of carbon dioxide and water to bicarbonate and protons by carbonic anhydrase. This would increase the proton gradient between the calicoblastic ectoderm and the calcifying medium (CM), the layer in which precipitation of new aragonite occurs [10], [17]. If the Ca^2+^/H^+^ ATP-ases on the calicoblastic membranes are not able to cope with this increased gradient in terms of proton removal from the CM, this would temporarily decrease its pH and aragonite saturation state, resulting in a reduction of calcification rates (Figure 2). To confirm this mechanism, pH micro sensor studies such as those described by Al-Horani et al. [17] should be conducted during feeding experiments. This allows for measuring changes in the pH of the CM during feeding, which could be used to infer changes in its aragonite saturation state. The fact that Szmant-Froelich and Pilson [30] found a pronounced increase (approximately 2.5-fold) in respiration rates of the coral *Astrangia danae* immediately after feeding on *Artemia* lends credence to this hypothesis. Tissue acidosis may induce a transient energy reallocation to processes other than calcification, including soft tissue growth and organic matrix synthesis, as this may be more energetically favourable.

Future studies may determine the threshold zooplankton concentration or zooplankton to coral biomass ratio below which no short-term reduction of dark calcification can be detected. Another issue which should be addressed is how long the inhibitory effect of heterotrophy lasts, which is likely to be only several hours when taking feeding and digestion rates of *G. fascicularis* into account [31], [32]. A temporal effect would explain the discrepancy between the inhibitory short-term [11,12, this paper] and enhancing long-term [1] effects of heterotrophy on coral calcification. Although dark calcification is temporarily inhibited during zooplankton feeding, in between feeding events, corals can benefit from enhanced organic matrix synthesis [2], [3], [4], photosynthesis rates [4], [5], [6], [7], [8], [9] and metabolic DIC supply [3], [10] which promote calcification. In this perspective, the nocturnal feeding behaviour of corals, possibly an adaptation strategy to higher zooplankton availability ([1] and references therein), may impose a significant physiological cost to corals in terms of impaired dark calcification rates. In addition, our results suggest that feeding scleractinian corals in aquaculture during daytime (i.e. in light) may be more optimal to growth.

In conclusion, this study demonstrates that oxygen is a key factor controlling calcification of scleractinian corals. However, oxygen limitation is most likely not the causal factor underlying the inhibitory short-term effect of heterotrophy on dark calcification. Temporal energy reallocation induced by tissue acidosis may explain this phenomenon. These insights enhance our understanding of how oxygen and heterotrophy affect coral calcification, both *in situ* as well as in aquaculture.

## Materials and Methods

### Ethics Statement

Captive bred corals (under CITES no. 52139) were provided by Burgers’ Zoo BV (Arnhem, The Netherlands). All experiments were conducted at Wageningen University (Wageningen, The Netherlands). No approval from an ethics committee was required as scleractinian corals are exempted from legislation concerning the use of animals for scientific purposes in the European Union (Directive 2010/63/EU).

### Selected Species and Husbandry

For this study, we used the Indo-Pacific scleractinian species *Galaxea fascicularis* (Linnaeus 1767). All colonies were genetically identical as they originated from the same parent colony. Corals were kept in a closed system of 400 L. Water flow was provided by three Turbelle nanostream 6045 circulation pumps (Tunze Aquarientechnik GmbH, Penzberg, Germany) providing a total flow rate of 13,500 L h^−1^. Water parameters were maintained at the following levels: salinity 35.0±0.3 g L^−1^, temperature 26±0.5°C, pH 8.2±0.3, a quantum irradiance (QI) of 170 µmol m^−2^ s^−1^ (12/12 h light regime), ammonium 0.01±0.01 mg L^−1^, nitrate 0.13±0.03 mg L^−1^, phosphate 0.02±0.01 mg L^−1^, calcium 400±25 mg L^−1^, magnesium 1300±60 mg L^−1^.

### Analysis of Colony Surface Area and Polyp Number

To determine projected surface area and polyp number, colonies (n = 4) were removed from the aquarium and photographed directly from above, together with a ruler. A HDR-CX505VE digital camera (Sony Corporation, Tokyo, Japan) was used to record images. Projected surface area was determined by image analysis using ImageTool 3.0 every two weeks, during which the live circumference of the colonies was traced. Surface area was calculated by using the ruler as a reference and was expressed in cm^2^. Polyp number was determined by marking individual polyps using the count function of the software. To prevent stress-induced artifacts, surface area and polyp number were never measured before treatments.

### Analysis of Colony Volume

Water displacement was used to determine colony volume. Drip-dry corals were submerged in 500 mL seawater in 800 mL beakers after which the displaced water was transferred and measured in graduated cylinders and expressed in mL.

### Calcification Measurements

To measure calcification rates for *G. fascicularis*, we used the alkalinity anomaly technique [33]. Colonies with a starting size of 30.37±4.56 cm^2^ and polyp count of 164±15 polyps (n = 4) were incubated in cells with a gross volume of 1547±3 ml for 6 hours. Net water volumes were calculated by subtracting total volumes of all objects in the cells from the gross cell volumes, including colony volumes. To determine the short-term effects of heterotrophy on light and dark calcification rates under a wide range of oxygen saturations, all 4 colonies were subjected to a total of 24 different treatments in a randomised factorial repeated measures design that were carried out over a four-month period. Treatments were light (QI of 250 µmol m^−2^ s^−1^) or complete darkness (2 levels), with or without 150 *Artemia* nauplii per coral polyp (2 levels), at ambient oxygen saturations of 13, 50, 80, 110, 150 and 280% (or 0.87; 3.33; 5.33; 7.33; 10.00 and 18.67 mg L^−1^ O_2_, respectively, 6 levels). The QI was chosen to saturate zooxanthellae photosynthesis, thereby preventing a possible light limitation which could obscure the (interactive) effect of light [34]. The prey dosage was chosen in order to reflect aquaculture conditions, and to ensure that sufficient feeding events would occur during the short incubations. To maintain stable oxygen saturations during the entire incubations, five 5850S smart flow mass controllers (Brooks International, Hatfield, USA) were connected to two digital microprocessor units, models 0152/0154 (Brooks International, Hatfield, USA) which allowed for controlling volumetric flow rates of various gases in each cell. Nitrogen gas (N_2_) was used for the 13, 50 and 80% oxygen saturation treatments. Compressed air was used for the 110% treatment. Pure oxygen (O_2_) was used to for the 150 and 280% treatments. Oxygen concentrations were monitored throughout all experiments with IntelliCAL™ LDO101 luminescent dissolved oxygen probes (Hach-Lange GmbH, Düsseldorf, Germany). *Artemia* nauplii (average nauplii length was 440 µm) were hatched from cysts (Great Salt Lake Artemia cysts, Artemia International LLC, Fairview, USA) at a salinity of 25 g L^−1^ and a temperature of 28°C, and used immediately after hatching. The daily concentrations of *Artemia* cultures were determined by counting three seawater-diluted (1∶99 mL) aliquots under an M8 stereomicroscope (Wild Heerbrugg, Heerbrugg, Switzerland), and used to calculate required volumes to obtain a dosage of 150 nauplii per polyp. Temperature was kept at 26±0.5°C by means of water jackets surrounding each incubation chamber, which were connected to a water bath equipped with a TC20 water cooler (Teco SRL, Ravenna, Italy). Water flow was provided with magnetic stirring plates (IKA Werke GmbH & Co. KG, Staufen, Germany), and was estimated at approximately 5 cm s^−1^. Water from the maintenance system was used to fill the incubation chambers, to minimise stress to the coral colonies. Calcium and alkalinity are known to influence calcification rates [35], [36], and were always measured and adjusted when required to 400 mg L^−1^ and 2.50 mEq L^−1^, respectively, before every experiment. Two water samples of 50 mL each were taken from every incubation chamber at t = 0 and t = 6 hours for determination of total alkalinity (A_T_) and inorganic nutrients. This was taken into account during calculation of net cell volumes. During feeding treatments, water samples were filtered on a sterile filter mesh (150 µm pore size) to remove nauplii before measurement. To determine A_T_, 50 ml samples were potentiometrically titrated on a Titralab 840 (Radiometer Analytical SAS, Lyon, France) with 0.02 M HCl to inflection point. Changes in A_T_, expressed in mEq L^−1^, were calculated for each cell. During each experiment, a control cell containing only the same seawater was used, except for feeding experiments where the average amount of *Artemia* nauplii dosed to the coral cells was included. Background alkalinity changes were used to correct all data.

Inorganic nutrients are known to influence alkalinity, and can therefore be a source of artifacts in the alkalinity anomaly technique [37]. To correct for changes in inorganic nutrient concentrations and therefore A_T_, we measured ammonia (NH_3_) and orthophosphate (PO_4_^3−^) concentrations during all experiments at t = 0 and t = 6 hours with a seawater calibrated DR 2800 spectrophotometer (Hach-Lange GmbH, Düsseldorf, Germany). Changes in NH_3_ and PO_4_^3−^ concentrations were converted to alkalinity changes in mEq L^−1^ and subsequently used to correct all data, including controls. We used mmol L^−1^ to mEq L^−1^ ratios of 1∶1 and 1∶3 for NH_3_ and PO_4_^3−^, respectively. Total A_T_ depletions in mEq were calculated by taking net cell volumes into account. These were subsequently converted to mg calcium carbonate (CaCO_3_) fixed, by using a mEq to mg CaCO_3_ ratio of 1∶50.04. Differences in coral biomass, and related to that the amount of *Artemia* nauplii fed, were taken into account by expressing all data as mg CaCO_3_ per cm^2^ coral tissue per hour. Between incubations, we incorporated resting periods lasting at least 48 hours to minimise artifacts due to stress caused by the experiments. All corals were acclimated to each experimental condition for 15 minutes before the start of every experiment, i.e. t = 0 was defined as the time point directly following the acclimation period. As calcification rates of *G. fascicularis* may vary during daytime [11], experiments were conducted within the same time interval of 9∶00 to 17∶00 hrs.

### Data Analysis

Normality of data was evaluated by plotting residuals of each dataset versus predicted values, and by performing a Shapiro-Wilk test. All data were found to be normally distributed (p>0.050). As sphericity for oxygen could not be calculated using Machly’s test, we used a conservative Greenhouse-Geisser correction to adjust the degrees of freedom for oxygen and its interactions with light and feeding. We used a three-way factorial ANOVA for repeated measures, followed by Bonferroni’s test for *post-hoc* analysis of oxygen treatments. Simple effects were used to elucidate interactive effects. Statistical analysis was performed with IBM SPSS Statistics 19 (IBM Corp., Armonk, USA). Graphs were plotted with SigmaPlot 11.0 (Systat Software, Inc., San Jose, USA). All data presented are means ± s.d.
